# p21cip1/waf1 Coordinates Autophagy, Proliferation and Apoptosis in Response to Metabolic Stress

**DOI:** 10.3390/cancers11081112

**Published:** 2019-08-03

**Authors:** Kanjoormana Aryan Manu, Pham Hong Anh Cao, Tin Fan Chai, Patrick J. Casey, Mei Wang

**Affiliations:** 1 Program in Cancer and Stem Cell Biology, Duke-NUS Graduate Medical School, Singapore 169857, Singapore; 2 Department of Pharmacology and Cancer Biology, Duke University School of Medicine, Durham, NC 27710, USA; 3 Department of Biochemistry, National University of Singapore, Singapore 117596, Singapore

**Keywords:** p21, metabolic stress, cell cycle, autophagy, apoptosis, cancer

## Abstract

Cancer cells possess metabolic properties that are different from benign cells. These unique characteristics have become attractive targets that are being actively investigated for cancer therapy. p21cip1/waf1, also known as Cyclin-Dependent Kinase inhibitor 1A, is encoded by the *CDKN1A* gene. It is a major p53 target gene involved in cell cycle progression that has been extensively evaluated. To date, p21 has been reported to regulate various cell functions, both dependent and independent of p53. Besides regulating the cell cycle, p21 also modulates apoptosis, induces senescence, and maintains cellular quiescence in response to various stimuli. p21 transcription is induced in response to stresses, including those from oxidative and chemotherapeutic treatment. A recent study has shown that in response to metabolic stresses such as nutrient and energy depletion, p21 expression is induced to regulate various cell functions. Despite the biological significance, the mechanism of p21 regulation in cancer adaptation to metabolic stress is underexplored and thus represents an exciting field. This review focuses on the recent development of p21 regulation in response to metabolic stress and its impact in inducing cell cycle arrest and death in cancer cells.

## 1. Introduction

Eukaryotic cells utilize complicated mechanisms to regulate cell division and quiescence in response to both internal and external stimuli. These cells rapidly divide under certain conditions such as embryonic development and wound healing. Conversely, they stop proliferating in response to adverse conditions and enter cell cycle arrest and post-development quiescence. Hence, the cells develop a sophisticated braking system required for survival. Deregulation of this mechanism can result in loss of genome integrity and cancer development. p21 (Cip1/WAF1) was first identified as a Cyclin-Dependent Kinase (CDK) regulator that inhibits the retinoblastoma gene (Rb) phosphorylation and G1/S cell cycle progression [1]. In this capacity, p21 was identified as the p53 target gene that suppresses the growth of human brain, lung and colon tumor cells in vitro [2]. Early studies also discovered that interactions between p21 and other proteins, most notably CDKs, are highly regulated events that control cell cycle progression [3,4,5]. p21 binds to CDK and obstructs CDK interaction with its substrates such as the Rb family members, hence, negatively regulating G1/S cell cycle progression [6,7,8]. Following DNA damage, p21 prevents Cdc25 activation by competing with its binding to PCNA (Proliferating Cell Nuclear Antigen), thus maintaining G2/M arrest [9,10]. Since its discovery over 25 years ago, p21 has been characterized as an important player that employs different mechanisms to regulate multiple cellular functions. As evident by the growing literature, p21 regulation continues to attract significant attention from various researchers in many fields.

## 2. Regulation of p21

### 2.1. Transcriptional Regulation of p21

p21 transcription can be regulated by either a p53-dependent or -independent manner. These regulations were described in the early years of p21 studies, where p21 induction in response to radiation-induced DNA damage was characterized as p53-dependent [11]. In contrast, its induction during differentiation was independent of p53 [12]. The cellular contexts on how p21 is regulated by these mechanisms remain an active field of study. Various studies have identified key players that interact with p53 to regulate p21 expression. These include NF-κB-p65 nuclear factor [13], the bZIP transcription factor (Zta) and NDF (Nucleosome-Destabilizing Factor) [14], BRCA1 (Breast Cancer type 1), p33ING1 (Inhibitor of Growth Family Member 1), p300/CBP (CREB Binding Protein) and IRF-1 (interferon regulatory factor 1) [15,16,17,18]. In addition, HRas also induces p21 transcription, where the HRas-ARF (ADP Ribosylation Factors)-p53-p21 circuit has been reported to induce senescence [19].

During normal tissue development and serum-stimulated growth in vitro, p21 is primarily regulated in a p53-independent manner by a number of transcription factors [20]. Under various growth conditions and cell types, different transcription factors have been identified to regulate p21 expression [21,22]. They include SMAD transcription factors downstream of Transforming Growth Factor-β (TGF-β) [23,24], Specificity Protein 1 (SP-1) [25], Myogenic Differentiation (MyoD) [26,27], BETA2 [28], progesterone receptors (PR) [29], and transcription factors AP2, E2Fs, C/EBPα, and C/EBPβ [14,30]. Increased Raf kinase expression also induces p21 and cell cycle arrest through the p53-independent pathway [14,30,31,32]. Different repressors have also been reported to regulate p21 expression. For example, Gfi-1B, a cellular proto-oncogene expressed in the bone marrow and spleen, is a direct repressor of the p21 promoter [33]. Another protein, HMG-Box Protein 1 (HBP1), can inhibit E2F-stimulated p21 transcription [34]. Notably, MYC (MYC oncogene) can inhibit p21 transcription and contribute to anti-estrogen therapy resistance in ER-α-positive breast cancers [35]. Additionally, MYC induces AP4, another p21 repressor to inhibit p21 transcription [36].

Epigenetic processes can also regulate p21 transcriptional activation. They can be induced through the p53-independent pathway by chromatin remodeling following acetylation of histones H3 and H4 in the p21 promoter region [37,38,39,40]. DNA methylation also plays a role in p21 transcription. Hypermethylation of the promoter region near the Sp1 consensus element significantly reduces Sp1/Sp3 binding, thereby inhibiting p21 expression [41]. MYC also mediates the recruitment of DNA CpG methyltransferase 3a (Dnmt3a) to form a DNA binding complex and repress p21 expression [42]. In conclusion, there are complex mechanisms involved in the regulation of p21 transcription, which underscores its importance in cell function. Future studies will provide insights in contexts of p21 expression under physiological and pathophysiological conditions.

### 2.2. Post-Translational Regulation of p21

p21 is also subjected to post-translational modification. Most notably, it is regulated by serine-threonine kinases phosphorylation at Ser-130, Ser-146, Thr-57 and Thr-145. These phosphorylation events impact both the function and stability of p21. For example, Thr-145 phosphorylation by AKT kinase disrupts p21 binding to PCNA and enhances its cytoplasmic localization. This, in turn, promotes cell survival and transformation in breast cancers [43,44,45,46,47]. AKT phosphorylation at Ser-146 is also reported to increase p21 stability and cell survival [43]. Phosphorylation at Thr-57 and Ser-130 by Glycogen Synthase Kinase 3-β (GSK3β) and CDK2-Cyclin E, respectively, increases p21 ubiquitination and proteolytic degradation [48,49]. Although various phosphorylation events have been reported, their impact on p21 function has not all been clearly defined [30,50,51]. Thus, further investigation is necessary to enhance our understanding of how these post-translational modifications regulate p21 degradation and functions.

## 3. Cellular Functions of p21 in Normal and Cancer Cells

### 3.1. Regulating Cell Cycle Progression

Inhibition of cell cycle progression is one of the main cellular functions of p21. It mainly occurs by inhibiting cyclins A/CDK2, E/CDK2, and D1/CDK4 activities, thereby preventing the phosphorylation of Rb protein [52]. The ability to induce cell cycle arrest in response to various stresses defines the importance of p21 in the regulation of proliferation, particularly its role as a major tumor suppressor. The molecular mechanisms involved in this process have been extensively reviewed by others [30,53].

### 3.2. Mediating Apoptosis

p21 has been demonstrated to regulate apoptosis in a paradoxical manner, depending on the cellular context [30,54,55]. It has been implicated as a negative regulator for both p53-dependent and p53–independent apoptosis [53,56]. For example, loss of p21 sensitizes HCT116 cells to radiotherapy by enhancing apoptosis via the p53 pathway [57]. Independent of p53, p21 can inhibit apoptosis triggered by various signals such as TGF-β, Tumor Necrosis Factor- α (TNF-α), Interferon- γ (IFN-γ), and histone deacetylase inhibitors [53]. In contrast, numerous studies have shown a pro-apoptotic role of p21 in both p53-dependent and p53-independent manners. For example, p21 induction in mammary tumor cells enhances apoptosis independent of p53 activity [58]. Collectively, these studies have shown that p21 regulation of apoptosis and other functions involves complex mechanisms that are highly dependent on tissue differentiation and environmental stimuli [59,60,61].

### 3.3. Inducing Senescence

p21 is also a major regulator of cellular senescence, a complex program involving multiple signaling pathways. p21 interacts with p16INK4A tumor suppressor to inhibit cyclin-dependent kinases, suppressing retinoblastoma protein phosphorylation and the expression of cell proliferation-associated genes [62]. Similar to its regulation on other processes, p21’s impact on senescence occurs in both p53-dependent and –independent manners. For example, overexpressing p21 in p53-deficient cells promotes premature senescence and protects cancer cells from chemotherapeutic drugs [63]. A complex interaction between Reactive Oxygen Species (ROS) and p21 has also been reported to induce senescence and growth arrest [64]. Interestingly, p21-induced senescence was recently suggested as pro–survival in nature, as its suppression results in the death of senescent cells [65]. Similar to other processes, p21 regulation of senescence involves complex mechanisms that require in-depth investigations.

### 3.4. Maintaining Stem Cell Property

A hallmark of stem cells is their ability for self-renewal. Most stem cell populations are in the quiescent state until they are stimulated. p21 has been reported to maintain cellular quiescence [66,67] and postulated to function as a molecular switch for cell cycle entry. Absence of p21 leads to deregulated cell division and stem cell exhaustion. p53 has been reported to regulate cell cycle entry of hematopoietic stem cells [68]. However, p21 can be induced independently of p53, even acting to counter p53 modulation. For example, in response to irradiation-induced DNA damage, the p21-mediated induction of HES1 (Hairy and Enhancer of Split-1) could repress p53. This allows the stem cells to avoid apoptosis and preserves their ability to self-renew, countering p53 functioning [69,70]. In a p21 knockout mouse model, p21 promotes ALDH1 (Aldehyde Dehydrogenase-1) activity and tumor-initiating property partially through Wnt/TCF and cyclin D1 signaling pathways [71]. Although p21 is an active player in regulating both normal and cancer cell stemness, the associated signaling pathways remain to be clarified.

### 3.5. p21 in Cancer Metastasis

Apart from its well-established roles, p21 may also directly or indirectly influence cancer metastasis. However, the association between p21 expression and metastatic ability is inconsistent in different cancers. In some cancers, p21 levels are inversely related to the metastatic phenotype, where a higher p21 expression predicts a favorable disease outcome [72,73,74,75]. One possible explanation is that the loss of p21 reduces the inhibitory effect on cell cycle progression, enhancing the continuous proliferation independent of growth stimulatory signals. In contrast, high levels of p21 expression are associated with increased metastasis, the recurrence of disease and decreased patient survival in certain cancers [76,77,78]. One of the major hypothesis is that in addition to forming quaternary complexes with various cyclins/cdks, p21 accumulation predominantly forms binary complexes with PCNA [79]. Consequently, this releases active cyclin/cdk complexes that result in unfettered cellular proliferation [76]. Collectively, these studies indicate that the role of p21 in metastasis vary in different cancers. Therefore, more studies are required to delineate the precise underlying mechanism of p21 regulation in metastasis.

### 3.6. p21 in Cancer Therapy

Both p21 inactivation and activation have been implicated in cancer therapy resistance. p21 inactivation is associated with paclitaxel and 5-Fluorouracil (5FU) resistance in noncancerous breast epithelial and colon cancer cells, respectively [80,81]. In contrast, the induction of p21 expression is associated with a significant increase in the treatment resistance to paclitaxel and cisplatin in certain epidermoid carcinoma cells [82]. In another study, the STAT3-mediated transcriptional activation of p21 confers resistance to breast cancer cells against Taxol treatment [83]. In addition to its expression level, p21 localization also contributes to the cancer cell resistance to therapy. For instance, cytoplasmic p21 was shown to protect testicular embryonal carcinoma cells against cisplatin-induced apoptosis, while p21 translocation to the nucleus by AKT inhibition sensitizes cells to cisplatin [84]. Similarly, cytoplasmic p21 is accountable for 5FU resistance in colorectal cancers by inhibiting apoptosis [85]. Altogether, these findings suggest cytoplasmic p21 accumulation is associated with poor prognosis. Thus, in addition to its expression level, the regulation of p21 cellular localization should be taken into consideration in improving treatment response.

## 4. p21 Regulation in Response to Metabolic Stress

Metabolism is a cornerstone for various cellular functions. It is so important that Major metabolic enzymes and regulatory pathways are conserved from unicellular organisms to mammals. Metabolic stress can be triggered by either nutrient unavailability or malfunction of important proteins involved in the metabolic machinery. Adaption to metabolic stress is essential for cell proliferation and survival. p21 is involved in cellular responses to various stressors [86,87,88]. Recent findings on p21 responses to metabolic stress, its impact on cell proliferation and survival, as well as their relevance to cancer biology are discussed below.

### 4.1. p21 and Fasting

p21 plays an important role in cell response to nutrient deficiency. Studies have shown that p21 is transcriptionally upregulated in mice under short-term fasting [89,90]. A major mechanism involves the Forkhead Box O family of transcription factors, known regulators for gluconeogenesis, lipogenesis and autophagy. These factors bind to the p21 promoter and regulate its transcription during fasting [89,91,92]. In a p21 knockout mouse model, fasting resulted in reduced serum free fatty acids (FFA) and triglyceride (TG) levels. Compared with their wild-type littermates, the accumulation of ketone bodies occurs more rapidly in the knockout mice, suggesting a faster depletion of lipid storage [90]. Mice lacking p21 also showed muscle protein degradation during fasting, likely through a ubiquitination-related process [90]. Transcriptome analysis of p21 knockout mice after 24-hr fasting showed a defective activation of PPARα (Peroxisome Proliferator-Activated Receptor-α), a crucial protein for fasting adaptation, suggesting a link between p21 and PPARα in modulating physiological responses to fasting [90,93,94]. It is evident from the study that p21 plays an active role in regulating organismal responses to nutrient depletion.

### 4.2. p21 and AMPK

Cellular nutrient uptake can stimulate intracellular nutrient-sensing mechanisms. This, in turn, activates anabolic pathways aiming at increasing biosynthesis. Apart from nutrient availability, the stimulation of growth factor signaling also accelerates anabolism. Nutrient deprivation, particularly glucose, is sensed by the AMP-activated kinase (AMPK), a key regulator of the catabolic-anabolic balance. AMPK is an allosteric enzyme that is directly regulated by the AMP to ATP ratio, which is a readout for cellular energy status [95,96]. Given its key role, it is not surprising that the G1/S transition is directly regulated by glucose availability and AMPK activity [97]. The expression of a constitutively-active AMPKα2 catalytic subunit (CA-AMPK) in mouse embryonic fibroblasts (MEFs) significantly inhibited G1 to S-phase entry in the cell cycle. Interestingly, a similar expression of constitutively-active AMPK in p53-deficient MEFs failed to induce full cell cycle arrest. In subsequent studies, AMPK was found to phosphorylate p53 and activated its transcription activity, resulting in the upregulation of p21 protein production [97]. This suggests that the AMPK-p21 signaling pathways are involved in response to starvation. This finding was validated in a separate study that linked AMPK and p21 in regulating the cell cycle progression [98].

While it is not the focus of the current review, it is important to note that AMPK can also sense DNA damage stress and induce p21 transcription in both p53-dependent and-independent manners [99,100,101]. It appears that the activation of AMPK in this context is likely mediated through its upstream regulator LKB1 (Liver Kinase B1) [100,102,103]. In addition to AMPK, an AMPK-related kinase involved in metabolic homeostasis, MPK38 (Murine protein serine-threonine kinase 38 or Maternal embryonic leucine zipper kinase-MELK), was recently shown to interact with and stabilize p21, leading to p21-mediated apoptosis and cell cycle arrest [104]. Together, the evidence links AMPK as a central cell energy-sensing molecule in controlling p21 expression via either p53-dependent or- independent mechanisms, suggesting p21 involvement in the cellular response to metabolic stress. Nevertheless, more studies are necessary to delineate the mechanisms of AMPK-dependent p21 activation under different conditions and cell states.

### 4.3. p21 and Amino Acid Deficiency

Amino acids are important building blocks and signaling intermediators. Their deficiency can alter many cellular metabolic functions that, in turn, regulate cell cycle progression and survival through complex regulations, including those involving p21 [105,106,107,108]. The translation of p21 was selectively upregulated by Integrated Stress Response kinase GCN2, a widely-studied sensor for amino acid deficiency through the phosphorylation of the eukaryotic translation initiation factor eIF2α [108]. In addition to regulating p21 translation, amino acid deprivation has been suggested to activate ERK1/2 (extracellular signal-regulated kinases 1 and 2), which increases p21 mRNA stability [109]. PI3K and mTOR signaling may also impact the level of p21 [109]. Interestingly, GCN-2 expression is elevated in several tumors, suggesting an amino acid deficient state in the tumor microenvironment [107]. In addition, the GCN2 mediated induction of p21 may play a role in inhibiting tumor cell proliferation and reducing tumor growth [108]. Together, these findings indicate that amino acid availability, through multiple signaling pathways, can regulate the p21 level that, in turn, impacts cell proliferation.

## 5. p21 in Cancer Metabolism and Therapy

In cancer, p21 is generally perceived as a tumor-suppressor protein. This hypothesis is supported by the observations that p21-null mice spontaneously developed tumors [110]. Additionally, many human cancers such as colorectal, cervical, head and neck, as well as small-cell lung cancers have reduced p21 expression [73,111,112,113,114]. However, p21 has also been postulated as a pro-tumorigenic protein. However, this theory is derived from the correlations among p21 levels, tumor grades and stages, rather than a direct causal relationship analysis [30,115].

### 5.1. Metabolic Characteristics of Cancer Cells

Compared with their benign counterparts, cancer cells have distinct metabolic characteristics that present therapeutic opportunities. One of the most recognized signatures of cancer metabolism is the Warburg effect, or aerobic glycolysis, which describes the preference of cancer cells using glycolysis over oxidative phosphorylation to metabolize glucose and derivatives for energy production. As a result of incomplete oxidation, most cancer cells consume glucose to generate more metabolic intermediates and lactic acid than benign cells [116,117]. Multiple oncogenic molecules promote glycolysis; these include mutant RAS, MYC, PI3K, and the loss of p53 tumor suppressor [118].

Despite their dependence on glycolysis in the metabolism, mitochondria respiration in cancer cells is important for generating energy and nucleic acid synthesis [119]. As a matter of fact, one can argue that the remaining respiration capacity becomes indispensable to cancer cells [120,121]. Hence, targeting mitochondrial respiration is a viable approach for selected cancer cells. In this regard, it has been suggested that cancer cells, which already have reduced oxidative phosphorylation, are sensitive to mitochondria inhibitors [122]. From this standpoint, an effective type 2 diabetes agent metformin has been the focus of intense investigation for cancer treatment and prevention [120,123]. Although the molecular mechanism(s) behind its therapeutic effect requires further clarification, recent studies demonstrate that metformin reduces tumorigenesis by inhibiting the mitochondrial complex I activity [123,124,125,126]. Both population and in vitro cellular studies have suggested that metformin should be further evaluated for its efficacy as an anti-cancer agent [127,128,129,130]. Indeed, various prospective clinical trials that investigate the utility of metformin for cancer treatment are ongoing. Besides metformin, new mitochondria respiration inhibitors are under development [131]. In addition to aerobic glycolysis and respiration adaptations, various metabolic changes have been reported in cancer cells, including the dominant expression of pyruvate kinase isozyme M2 (PKM2) in place of PKM1 [132], as well as genetic mutations in succinate dehydrogenase (SDH) [133] and isocitrate dehydrogenase (IDH) [134]. Given the scope of the review, we will focus on p21 regulated processes.

### 5.2. p21 in Cancer Metabolism and Its Therapeutic Implications

RAS mutation driven-oncogenesis accounts for one-third of human cancers. The relevance of the RAS mutation becomes even more apparent if the aberrancies in relevant pathways such as EGFR (Epidermal Growth Factor Receptor) mutations and MAPK (Mitogen-Activated Protein Kinases) signaling components are also taken into consideration [135,136,137]. RAS-driven signaling stimulates cell growth and proliferation. However, when extremely elevated, RAS signaling can induce cell cycle arrest and senescence through activated RAF/ERK signaling [138,139,140]. The RAS/RAF/ERK pathway can further induce cell cycle arrest and senescence through upregulating p21 in some cancer cells [141]. The association between this signaling pathway and p21 is supported by the observation that the manipulation of the post-translational modification enzyme isoprenylcysteine carboxylmethyltransferase (ICMT) activity up-regulates p21 levels [142]. ICMT is the last enzyme in the post-translational prenylation processing of CAAX proteins (containing C-terminus Cysteine-Aliphatic-Aliphatic-Any of a selection of amino acids), which include the RAS family of small GTPases. An early mouse model of RAS tumorigenesis has shown that ICMT loss of function up-regulates the level of p21 [143].

**Figure 1:**
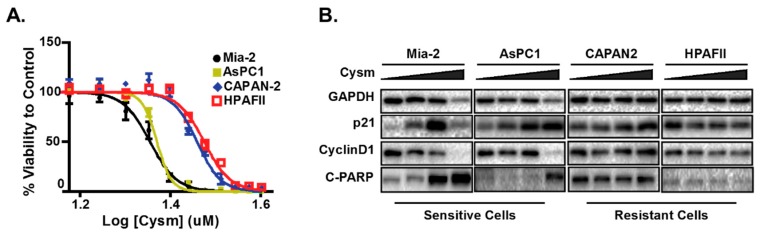
The pancreatic cell lines that are sensitive but not resistant to the isoprenylcysteine carboxylmethyltransferase (ICMT) inhibitor have elevated p21 and undergo apoptosis upon inhibitor treatment. (**A**) The ICMT inhibitor, named Cysmethynil (Cysm), dose-response curve of cell viability in different pancreatic cancer cell lines. (**B**) Immunoblots of sensitive and resistant cell lines indicating the expression of p21, cyclin D1 and apoptosis marker cleaved PARP (C-PARP, Poly-ADP-Ribose Polymerase 1). The figure is adopted from the published work by Manu et al., 2017 [142].

To better understand the mechanisms underlying p21 elevation in response to ICMT inhibition and its role in cancer cell proliferation and survival, we evaluated a panel of cancer cells from diverse tissue origins. Cancer cells that are sensitive to ICMT inhibition show a constellation of changes related to metabolism that include the reduction of mitochondria respiration, the induction of autophagy, cell cycle arrest and apoptosis [144,145,146,147]. Noteworthy, the p21 induction appears to accompany these phenotypes in sensitive cell lines (Figure 1) [142]. The treatment of cells with an ICMT inhibitor suppresses cellular proliferation and induces autophagy, both in in vitro and in vivo settings [146,148,149]. In addition to ICMT-induced metabolism changes observed above, the involvement of p21 in autophagy regulation, cell proliferation and survival was investigated [150,151].

To better understand how p21 regulation leads to cell adaptation following metabolic distress, it is necessary to briefly discuss the cellular process of autophagy—a fundamental eukaryotic cell adaptation in response to a nutrient-depleted state. The double membranous structure of autophagosomes forms as a self-digestive organelle in the lysosomal pathway to degrade damaged/aged organelles and protein aggregates; this maintains homeostasis in cells under normal conditions [152]. Autophagy is also an important catabolic response under the regulation of multiple signaling pathways. Under nutritional stress, cells are programmed to reduce anabolic activity and upregulate autophagy to sustain survival [153,154,155,156,157]. When persisted, the prolonged depleted state would lead to cell death either in an autophagy-dependent or independent manner [154]. Abnormalities in autophagy regulation have been implicated in different disease states, including cancer [158]. Manipulating autophagy levels has recently been implemented in cancer treatment [159,160,161].

**Figure 2:**
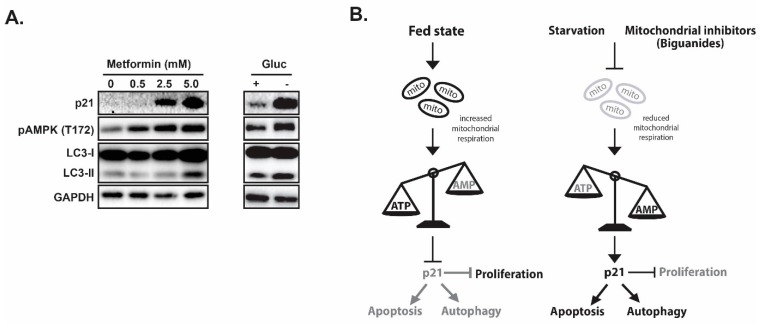
p21 coordinates autophagy, proliferation and apoptosis in response to metabolic stress. (**A**) Mitochondria inhibitor metformin treatment induces pAMPK, p21 and autophagy. (**B**) Proposed model of p21 as a coordinator for cell cycle, autophagy and apoptosis in response to nutrient/energy depletion.

In evaluating the impacts of ICMT inhibition in cancer cells, we found that p21 is induced only in the sensitive cell lines upon ICMT suppression. Marked reduction in mitochondrial respiration and consistent depletion of cellular “fuel molecules”, such as ATP and other NTPs, were also noted following exposures to the ICMT inhibitor. The mitochondria targeting effect has been shown to be responsible for most of the anti-proliferative effects [147]. The phenotypes of ICMT inhibition were further investigated in a panel of pancreatic cancer cell lines to compare responses from both treatment sensitive and resistant cells [142]. When ICMT was suppressed by either pharmacological or genetic means, both p21 mRNA and protein levels were significantly upregulated only in the sensitive pancreatic cancer cells. This was accompanied by reduced mitochondria respiration, cell cycle arrest and eventually cell death. Consistent with energy stress, ICMT inhibition in sensitive cells also resulted in AMPK activation. In contrast, lack of constellation of phenotypes in p21 induction, AMPK activation, autophagy, cell cycle arrest and cell death was observed in cancer cells that are resistant to the ICMT inhibitor. We hypothesize that if p21 mediates cell responses to nutrition and energy depletion, then glucose starvation or treatment with mitochondria inhibiting agents should elicit similar responses in these sensitive cells. Metformin, as we have discussed earlier, has been reported to inhibit the mitochondria respiratory chain complex I. Indeed, we have observed similar responses of p21 elevation, AMPK activation and other relevant phenotypes in cells that were exposed to either glucose starvation or metformin treatment (Figure 2A), lending strong support for the role of p21 in the regulation of cellular responses to metabolic stress in many cell types.

As discussed earlier, p21 regulates cell cycle progression and apoptosis. Therefore, it makes physiological sense that p21 would be involved in sensing and transmitting the signal of metabolic stress. Indeed, when we further evaluated the role of p21 in response to ICMT inhibition, we found that p21 is an upstream regulator of cell cycle progression, autophagy and apoptosis, through the transcriptional regulation of downstream effectors that include BNIP3 (BCL2 Interacting Protein 3), LC3 (Microtubule-associated protein 1A/1B light chain 3B) and ULK1 (Unc-51 Like Autophagy Activating Kinase 1). Both ULK1 and LC3 are well-known proteins that promote the initiation and extension of autophagosomes. BNIP3 is a mitochondrial member of proapoptotic BCL2 family proteins containing a motif similar to the BH3 domain, which is a regulator of both autophagy and apoptosis [162,163,164,165,166]. BNIP3 upregulation was reported to sensitize cancer cells to apoptosis, while the loss of BNIP3 rendered cancer cell more resistant towards therapy [167,168,169]. Consistent with its role in cancer cell death [170,171,172,173], it is no surprise that BNIP3 expression was suppressed in several types of cancers. The role of p21 in promoting autophagy and apoptosis was also evaluated by the concurrent knockdown of ICMT and p21. In ICMT inhibition-sensitive MiaPaCa-2 pancreatic cancer cells, the loss of p21 rescued the cell cycle arrest, autophagy and apoptosis that accompanied ICMT inhibition.

In summary, we have discussed the evidence that supports p21 as a key regulator and signaling node in mediating cancer cells’ response to nutrition and fuel depletion. Overall, the evidence supports that p21 functions to integrate catabolism activities, cell cycle progression, and ultimately programmed cell death (Figure 2B). While the exact underlying mechanism(s) are yet to be identified, it makes sense that cells would utilize p21 as a central biological coordinator to respond to energy starvation by (i) halting growth and proliferation as these activities require both building blocks and energy, (ii) increasing catabolic activity, such as autophagy, to produce fuels and metabolic intermediates to sustain essential cell activities for survival, and (iii) initiating the cell death process when faced with persistent metabolic stress. While more research is required to delineate the role of p21 in these processes, such knowledge would be highly applicable for the future development of cancer therapeutics and for biomarker identification.

## 6. Discussion

The field of p21 has grown tremendously in the last three decades. Since its discovery in the 1980s as a Cyclin-Dependent Kinase inhibitor, p21 has attracted significant attention from researchers in many fields, as evident from the thousands of research publications on this protein. To date, p21 is known not only as a cell cycle regulator, but also as a key player in various other cellular functions including senescence, apoptosis, and self-renewal. p21 has also been long regarded as a stress response protein. In this regard, p21 can function as a coordinator for DNA damage repair pathways. Adding to its portfolio, p21 has recently been reported to act as a central regulator in cell adaptation to metabolic stress, particularly energy depletion in response to either starvation or treatment with mitochondria respiration inhibitors. Although this aspect has only been recently investigated with relatively few publications, the impact of these findings in understanding basic cell biology and the development of potential new therapy is apparent. For example, although recent findings demonstrate that stress-induced levels of p21 primarily leads to cell cycle arrest and cell death, the exact outcomes of p21 activation are very much dependent on the type, intensity and duration of particular stress, as well as the cell type upon which the stress acts [142]. Another example is the role of p21 in maintaining stemness. p21 has been reported to help stem cells maintain quiescence and genomic integrity, and possibly inhibit apoptosis. However, during prolonged stress, p21 can activate apoptosis. Therefore, it will be an interesting, albeit challenging field of research, to further define the role and impact of p21 regulation in the context of different stimuli and cell types.

This review hopefully highlights (i) the significance of emerging p21 research as a promising therapeutic approach for biologists and oncologists, and (ii) the necessity to continue the effort in understanding p21’s function as a coordinator of metabolic stress responses. As mentioned above, because of its dichotomy function, it is a challenging task to fully understand how p21 regulates various biological and cellular functions, particularly those associated with the cancer metabolism and its potential therapeutic implementation. Concerted explorations to understand p21 in adaptation to stress, metabolic and other stimuli, are essential for its translational application in cancer therapy.

## References

[1] Harper J.W., Adami G.R., Wei N., Keyomarsi K., Elledge S.J. (1993). The p21 Cdk-interacting protein Cip1 is a potent inhibitor of G1 cyclin-dependent kinases. Cell.

[2] El-Deiry W.S., Tokino T., Velculescu V.E., Levy D.B., Parsons R., Trent J.M., Lin D., Mercer W.E., Kinzler K.W., Vogelstein B. (1993). WAF1, a potential mediator of p53 tumor suppression. Cell.

[3] Noda A., Ning Y., Venable S.F., Pereira-Smith O.M., Smith J.R. (1994). Cloning of senescent cell-derived inhibitors of DNA synthesis using an expression screen. Exp. Cell Res..

[4] Xiong Y., Hannon G.J., Zhang H., Casso D., Kobayashi R., Beach D. (1993). p21 is a universal inhibitor of cyclin kinases. Nature.

[5] Harper J.W., Elledge S.J., Keyomarsi K., Dynlacht B., Tsai L.H., Zhang P., Dobrowolski S., Bai C., Connell-Crowley L., Swindell E. (1995). Inhibition of cyclin-dependent kinases by p21. Mol. Biol. Cell.

[6] Saha P., Eichbaum Q., Silberman E.D., Mayer B.J., Dutta A. (1997). p21CIP1 and Cdc25A: Competition between an inhibitor and an activator of cyclin-dependent kinases. Mol. Biol. Cell.

[7] Shiyanov P., Bagchi S., Adami G., Kokontis J., Hay N., Arroyo M., Morozov A., Raychaudhuri P. (1996). p21 Disrupts the interaction between cdk2 and the E2F-p130 complex. Mol. Cell. Biol..

[8] Zhu L., Harlow E., Dynlacht B.D. (1995). p107 uses a p21CIP1-related domain to bind cyclin/cdk2 and regulate interactions with E2F. Genes Dev..

[9] Ando T., Kawabe T., Ohara H., Ducommun B., Itoh M., Okamoto T. (2001). Involvement of the interaction between p21 and proliferating cell nuclear antigen for the maintenance of G2/M arrest after DNA damage. J. Biol. Chem..

[10] Gulbis J.M., Kelman Z., Hurwitz J., O’Donnell M., Kuriyan J. (1996). Structure of the C-terminal region of p21(WAF1/CIP1) complexed with human PCNA. Cell.

[11] El-Deiry W.S., Tokino T., Waldman T., Oliner J.D., Velculescu V.E., Burrell M., Hill D.E., Healy E., Rees J.L., Hamilton S.R. (1995). Topological control of p21WAF1/CIP1 expression in normal and neoplastic tissues. Cancer Res..

[12] Jiang H., Lin J., Su Z.Z., Collart F.R., Huberman E., Fisher P.B. (1994). Induction of differentiation in human promyelocytic HL-60 leukemia cells activates p21, WAF1/CIP1, expression in the absence of p53. Oncogene.

[13] Bash J., Zong W.X., Gelinas C. (1997). c-Rel arrests the proliferation of HeLa cells and affects critical regulators of the G1/S-phase transition. Mol. Cell. Biol..

[14] Gartel A.L., Tyner A.L. (1999). Transcriptional regulation of the p21((WAF1/CIP1)) gene. Exp. Cell Res..

[15] Zhang H., Somasundaram K., Peng Y., Tian H., Zhang H., Bi D., Weber B.L., El-Deiry W.S. (1998). BRCA1 physically associates with p53 and stimulates its transcriptional activity. Oncogene.

[16] Garkavtsev I., Grigorian I.A., Ossovskaya V.S., Chernov M.V., Chumakov P.M., Gudkov A.V. (1998). The candidate tumour suppressor p33ING1 cooperates with p53 in cell growth control. Nature.

[17] Lill N.L., Grossman S.R., Ginsberg D., DeCaprio J., Livingston D.M. (1997). Binding and modulation of p53 by p300/CBP coactivators. Nature.

[18] Tanaka N., Ishihara M., Lamphier M.S., Nozawa H., Matsuyama T., Mak T.W., Aizawa S., Tokino T., Oren M., Taniguchi T. (1996). Cooperation of the tumour suppressors IRF-1 and p53 in response to DNA damage. Nature.

[19] Swarbrick A., Roy E., Allen T., Bishop J.M. (2008). Id1 cooperates with oncogenic Ras to induce metastatic mammary carcinoma by subversion of the cellular senescence response. Proc. Natl. Acad. Sci. USA.

[20] Macleod K.F., Sherry N., Hannon G., Beach D., Tokino T., Kinzler K., Vogelstein B., Jacks T. (1995). p53-dependent and independent expression of p21 during cell growth, differentiation, and DNA damage. Genes Dev..

[21] Kennett S.B., Udvadia A.J., Horowitz J.M. (1997). Sp3 encodes multiple proteins that differ in their capacity to stimulate or repress transcription. Nucleic Acids Res..

[22] Somasundaram K., Zhang H., Zeng Y.X., Houvras Y., Peng Y., Zhang H., Wu G.S., Licht J.D., Weber B.L., El-Deiry W.S. (1997). Arrest of the cell cycle by the tumour-suppressor BRCA1 requires the CDK-inhibitor p21WAF1/CiP1. Nature.

[23] Li J.M., Datto M.B., Shen X., Hu P.P., Yu Y., Wang X.F. (1998). Sp1, but not Sp3, functions to mediate promoter activation by TGF-beta through canonical Sp1 binding sites. Nucleic Acids Res..

[24] Moustakas A., Kardassis D. (1998). Regulation of the human p21/WAF1/Cip1 promoter in hepatic cells by functional interactions between Sp1 and Smad family members. Proc. Natl. Acad. Sci. USA.

[25] Biggs J.R., Kudlow J.E., Kraft A.S. (1996). The role of the transcription factor Sp1 in regulating the expression of the WAF1/CIP1 gene in U937 leukemic cells. J. Biol. Chem..

[26] Halevy O., Novitch B.G., Spicer D.B., Skapek S.X., Rhee J., Hannon G.J., Beach D., Lassar A.B. (1995). Correlation of terminal cell cycle arrest of skeletal muscle with induction of p21 by MyoD. Science.

[27] Puri P.L., Avantaggiati M.L., Balsano C., Sang N., Graessmann A., Giordano A., Levrero M. (1997). p300 is required for MyoD-dependent cell cycle arrest and muscle-specific gene transcription. EMBO J..

[28] Mutoh H., Naya F.J., Tsai M.J., Leiter A.B. (1998). The basic helix-loop-helix protein BETA2 interacts with p300 to coordinate differentiation of secretin-expressing enteroendocrine cells. Genes Dev..

[29] Owen G.I., Richer J.K., Tung L., Takimoto G., Horwitz K.B. (1998). Progesterone regulates transcription of the p21(WAF1) cyclin- dependent kinase inhibitor gene through Sp1 and CBP/p300. J. Biol. Chem..

[30] Abbas T., Dutta A. (2009). p21 in cancer: Intricate networks and multiple activities. Nat. Rev. Cancer.

[31] Sewing A., Wiseman B., Lloyd A.C., Land H. (1997). High-intensity Raf signal causes cell cycle arrest mediated by p21Cip1. Mol. Cell. Biol..

[32] Pumiglia K.M., Decker S.J. (1997). Cell cycle arrest mediated by the MEK/mitogen-activated protein kinase pathway. Proc. Natl. Acad. Sci. USA.

[33] Tong B., Grimes H.L., Yang T.Y., Bear S.E., Qin Z., Du K., El-Deiry W.S., Tsichlis P.N. (1998). The Gfi-1B proto-oncoprotein represses p21WAF1 and inhibits myeloid cell differentiation. Mol. Cell. Biol..

[34] Gartel A.L., Goufman E., Tevosian S.G., Shih H., Yee A.S., Tyner A.L. (1998). Activation and repression of p21(WAF1/CIP1) transcription by RB binding proteins. Oncogene.

[35] Mukherjee S., Conrad S.E. (2005). c-Myc suppresses p21WAF1/CIP1 expression during estrogen signaling and antiestrogen resistance in human breast cancer cells. J. Biol. Chem..

[36] Jung P., Menssen A., Mayr D., Hermeking H. (2008). AP4 encodes a c-MYC-inducible repressor of p21. Proc. Natl. Acad. Sci. USA.

[37] Kim Y.B., Lee K.H., Sugita K., Yoshida M., Horinouchi S. (1999). Oxamflatin is a novel antitumor compound that inhibits mammalian histone deacetylase. Oncogene.

[38] Sambucetti L.C., Fischer D.D., Zabludoff S., Kwon P.O., Chamberlin H., Trogani N., Xu H., Cohen D. (1999). Histone deacetylase inhibition selectively alters the activity and expression of cell cycle proteins leading to specific chromatin acetylation and antiproliferative effects. J. Biol. Chem..

[39] Habold C., Poehlmann A., Bajbouj K., Hartig R., Korkmaz K.S., Roessner A., Schneider-Stock R. (2008). Trichostatin A causes p53 to switch oxidative-damaged colorectal cancer cells from cell cycle arrest into apoptosis. J. Cell. Mol. Med..

[40] Lagger G., Doetzlhofer A., Schuettengruber B., Haidweger E., Simboeck E., Tischler J., Chiocca S., Suske G., Rotheneder H., Wintersberger E. (2003). The tumor suppressor p53 and histone deacetylase 1 are antagonistic regulators of the cyclin-dependent kinase inhibitor p21/WAF1/CIP1 gene. Mol. Cell. Biol..

[41] Zhu W.G., Srinivasan K., Dai Z., Duan W., Druhan L.J., Ding H., Yee L., Villalona-Calero M.A., Plass C., Otterson G.A. (2003). Methylation of adjacent CpG sites affects Sp1/Sp3 binding and activity in the p21(Cip1) promoter. Mol. Cell. Biol..

[42] Brenner C., Deplus R., Didelot C., Loriot A., Vire E., De Smet C., Gutierrez A., Danovi D., Bernard D., Boon T. (2005). Myc represses transcription through recruitment of DNA methyltransferase corepressor. EMBO J..

[43] Li Y., Dowbenko D., Lasky L.A. (2002). AKT/PKB phosphorylation of p21Cip/WAF1 enhances protein stability of p21Cip/WAF1 and promotes cell survival. J. Biol. Chem..

[44] Rossig L., Jadidi A.S., Urbich C., Badorff C., Zeiher A.M., Dimmeler S. (2001). Akt-dependent phosphorylation of p21(Cip1) regulates PCNA binding and proliferation of endothelial cells. Mol. Cell. Biol..

[45] Zhou B.P., Liao Y., Xia W., Spohn B., Lee M.H., Hung M.C. (2001). Cytoplasmic localization of p21Cip1/WAF1 by Akt-induced phosphorylation in HER-2/neu-overexpressing cells. Nat. Cell Biol..

[46] Winters Z.E., Leek R.D., Bradburn M.J., Norbury C.J., Harris A.L. (2003). Cytoplasmic p21WAF1/CIP1 expression is correlated with HER-2/neu in breast cancer and is an independent predictor of prognosis. Breast Cancer Res..

[47] Xia W., Chen J.S., Zhou X., Sun P.R., Lee D.F., Liao Y., Zhou B.P., Hung M.C. (2004). Phosphorylation/cytoplasmic localization of p21Cip1/WAF1 is associated with HER2/neu overexpression and provides a novel combination predictor for poor prognosis in breast cancer patients. Clin. Cancer Res..

[48] Bornstein G., Bloom J., Sitry-Shevah D., Nakayama K., Pagano M., Hershko A. (2003). Role of the SCFSkp2 ubiquitin ligase in the degradation of p21Cip1 in S phase. J. Biol. Chem..

[49] Rossig L., Badorff C., Holzmann Y., Zeiher A.M., Dimmeler S. (2002). Glycogen synthase kinase-3 couples AKT-dependent signaling to the regulation of p21Cip1 degradation. J. Biol. Chem..

[50] Oh Y.T., Chun K.H., Park B.D., Choi J.S., Lee S.K. (2007). Regulation of cyclin-dependent kinase inhibitor p21WAF1/CIP1 by protein kinase Cdelta-mediated phosphorylation. Apoptosis.

[51] Scott M.T., Ingram A., Ball K.L. (2002). PDK1-dependent activation of atypical PKC leads to degradation of the p21 tumour modifier protein. EMBO J..

[52] Karimian A., Ahmadi Y., Yousefi B. (2016). Multiple functions of p21 in cell cycle, apoptosis and transcriptional regulation after DNA damage. DNA Repair (Amst.).

[53] Gartel A.L., Tyner A.L. (2002). The role of the cyclin-dependent kinase inhibitor p21 in apoptosis. Mol. Cancer Ther..

[54] Seoane J., Le H.V., Massague J. (2002). Myc suppression of the p21(Cip1) Cdk inhibitor influences the outcome of the p53 response to DNA damage. Nature.

[55] Kokontis J.M., Wagner A.J., O’Leary M., Liao S., Hay N. (2001). A transcriptional activation function of p53 is dispensable for and inhibitory of its apoptotic function. Oncogene.

[56] Waldman T., Lengauer C., Kinzler K.W., Vogelstein B. (1996). Uncoupling of S phase and mitosis induced by anticancer agents in cells lacking p21. Nature.

[57] Wouters B.G., Giaccia A.J., Denko N.C., Brown J.M. (1997). Loss of p21Waf1/Cip1 sensitizes tumors to radiation by an apoptosis-independent mechanism. Cancer Res..

[58] Shibata M.A., Yoshidome K., Shibata E., Jorcyk C.L., Green J.E. (2001). Suppression of mammary carcinoma growth in vitro and in vivo by inducible expression of the Cdk inhibitor p21. Cancer Gene Ther..

[59] Tsao Y.P., Huang S.J., Chang J.L., Hsieh J.T., Pong R.C., Chen S.L. (1999). Adenovirus-mediated p21((WAF1/SDII/CIP1)) gene transfer induces apoptosis of human cervical cancer cell lines. J. Virol..

[60] Yang Z.Y., Perkins N.D., Ohno T., Nabel E.G., Nabel G.J. (1995). The p21 cyclin-dependent kinase inhibitor suppresses tumorigenicity in vivo. Nat. Med..

[61] Erhardt J.A., Pittman R.N. (1998). p21WAF1 induces permanent growth arrest and enhances differentiation, but does not alter apoptosis in PC12 cells. Oncogene.

[62] Collado M., Blasco M.A., Serrano M. (2007). Cellular senescence in cancer and aging. Cell.

[63] Wang Y., Blandino G., Givol D. (1999). Induced p21waf expression in H1299 cell line promotes cell senescence and protects against cytotoxic effect of radiation and doxorubicin. Oncogene.

[64] Macip S., Igarashi M., Fang L., Chen A., Pan Z.Q., Lee S.W., Aaronson S.A. (2002). Inhibition of p21-mediated ROS accumulation can rescue p21-induced senescence. EMBO J..

[65] Yosef R., Pilpel N., Papismadov N., Gal H., Ovadya Y., Vadai E., Miller S., Porat Z., Ben-Dor S., Krizhanovsky V. (2017). p21 maintains senescent cell viability under persistent DNA damage response by restraining JNK and caspase signaling. EMBO J..

[66] Nakanishi M., Adami G.R., Robetorye R.S., Noda A., Venable S.F., Dimitrov D., Pereira-Smith O.M., Smith J.R. (1995). Exit from G0 and entry into the cell cycle of cells expressing p21Sdi1 antisense RNA. Proc. Natl. Acad. Sci. USA.

[67] Cheng T., Rodrigues N., Shen H., Yang Y., Dombkowski D., Sykes M., Scadden D.T. (2000). Hematopoietic stem cell quiescence maintained by p21cip1/waf1. Science.

[68] Liu Y., Elf S.E., Miyata Y., Sashida G., Liu Y., Huang G., Di Giandomenico S., Lee J.M., Deblasio A., Menendez S. (2009). p53 regulates hematopoietic stem cell quiescence. Cell Stem Cell.

[69] Insinga A., Cicalese A., Faretta M., Gallo B., Albano L., Ronzoni S., Furia L., Viale A., Pelicci P.G. (2013). DNA damage in stem cells activates p21, inhibits p53, and induces symmetric self-renewing divisions. Proc. Natl. Acad. Sci. USA.

[70] Castella P., Sawai S., Nakao K., Wagner J.A., Caudy M. (2000). HES-1 repression of differentiation and proliferation in PC12 cells: Role for the helix 3-helix 4 domain in transcription repression. Mol. Cell. Biol..

[71] Benard O., Qian X., Liang H., Ren Z., Suyama K., Norton L., Hazan R.B. (2019). p21CIP1 Promotes Mammary Cancer-Initiating Cells via Activation of Wnt/TCF1/CyclinD1 Signaling. Mol. Cancer Res..

[72] Jiang M., Shao Z.M., Wu J., Lu J.S., Yu L.M., Yuan J.D., Han Q.X., Shen Z.Z., Fontana J.A. (1997). p21/waf1/cip1 and mdm-2 expression in breast carcinoma patients as related to prognosis. Int. J. Cancer.

[73] Bukholm I.K., Nesland J.M. (2000). Protein expression of p53, p21 (WAF1/CIP1), bcl-2, Bax, cyclin D1 and pRb in human colon carcinomas. Virchows Arch..

[74] Mitomi H., Mori A., Kanazawa H., Nishiyama Y., Ihara A., Otani Y., Sada M., Kobayashi K., Igarashi M. (2005). Venous invasion and down-regulation of p21(WAF1/CIP1) are associated with metastasis in colorectal carcinomas. Hepatogastroenterology.

[75] Bott S.R., Arya M., Kirby R.S., Williamson M. (2005). p21WAF1/CIP1 gene is inactivated in metastatic prostatic cancer cell lines by promoter methylation. Prostate Cancer Prostatic Dis..

[76] Erber R., Klein W., Andl T., Enders C., Born A.I., Conradt C., Bartek J., Bosch F.X. (1997). Aberrant p21(CIP1/WAF1) protein accumulation in head-and-neck cancer. Int. J. Cancer.

[77] Ferrandina G., Stoler A., Fagotti A., Fanfani F., Sacco R., De Pasqua A., Mancuso S., Scambia G. (2000). p21WAF1/CIP1 protein expression in primary ovarian cancer. Int. J. Oncol..

[78] Jeannon J.P., Soames J., Lunec J., Awwad S., Ashton V., Wilson J.A. (2000). Expression of cyclin-dependent kinase inhibitor p21(WAF1) and p53 tumour suppressor gene in laryngeal cancer. Clin. Otolaryngol. Allied Sci..

[79] Waga S., Hannon G.J., Beach D., Stillman B. (1994). The p21 inhibitor of cyclin-dependent kinases controls DNA replication by interaction with PCNA. Nature.

[80] Galmarini C.M., Bouchet B.P., Audoynaud C., Lamblot C., Falette N., Bertholon J., Wang Q., Beghin A., Dumontet C., Puisieux A. (2006). A p21/WAF1 mutation favors the appearance of drug resistance to paclitaxel in human noncancerous epithelial mammary cells. Int. J. Cancer.

[81] Zhang Y., Geng L., Talmon G., Wang J. (2015). MicroRNA-520g confers drug resistance by regulating p21 expression in colorectal cancer. J. Biol. Chem..

[82] Schmidt M., Fan Z. (2001). Protection against chemotherapy-induced cytotoxicity by cyclin-dependent kinase inhibitors (CKI) in CKI-responsive cells compared with CKI-unresponsive cells. Oncogene.

[83] Hawthorne V.S., Huang W.C., Neal C.L., Tseng L.M., Hung M.C., Yu D. (2009). ErbB2-mediated Src and signal transducer and activator of transcription 3 activation leads to transcriptional up-regulation of p21Cip1 and chemoresistance in breast cancer cells. Mol. Cancer Res..

[84] Koster R., di Pietro A., Timmer-Bosscha H., Gibcus J.H., van den Berg A., Suurmeijer A.J., Bischoff R., Gietema J.A., de Jong S. (2010). Cytoplasmic p21 expression levels determine cisplatin resistance in human testicular cancer. J. Clin. Investig..

[85] Maiuthed A., Ninsontia C., Erlenbach-Wuensch K., Ndreshkjana B., Muenzner J.K., Caliskan A., Husayn A.P., Chaotham C., Hartmann A., Vial Roehe A. (2018). Cytoplasmic p21 Mediates 5-Fluorouracil Resistance by Inhibiting Pro-Apoptotic Chk2. Cancers.

[86] Hoeferlin L.A., Oleinik N.V., Krupenko N.I., Krupenko S.A. (2011). Activation of p21-Dependent G1/G2 Arrest in the Absence of DNA Damage as an Antiapoptotic Response to Metabolic Stress. Genes Cancer.

[87] Kaija H.M., Sarkioja T., Kortelainen M.L., Vuoristo J.T., Huikuri H.V., Porvari K.S. (2012). Stress-specific responses of p21 expression: Implication of transcript variant p21 alt-a in long-term hypoxia. J. Cell. Biochem..

[88] Masgras I., Carrera S., de Verdier P.J., Brennan P., Majid A., Makhtar W., Tulchinsky E., Jones G.D., Roninson I.B., Macip S. (2012). Reactive oxygen species and mitochondrial sensitivity to oxidative stress determine induction of cancer cell death by p21. J. Biol. Chem..

[89] Tinkum K.L., White L.S., Marpegan L., Herzog E., Piwnica-Worms D., Piwnica-Worms H. (2013). Forkhead box O1 (FOXO1) protein, but not p53, contributes to robust induction of p21 expression in fasted mice. J. Biol. Chem..

[90] Lopez-Guadamillas E., Fernandez-Marcos P.J., Pantoja C., Munoz-Martin M., Martinez D., Gomez-Lopez G., Campos-Olivas R., Valverde A.M., Serrano M. (2016). p21(Cip1) plays a critical role in the physiological adaptation to fasting through activation of PPARalpha. Sci Rep..

[91] Zhang K., Li L., Qi Y., Zhu X., Gan B., DePinho R.A., Averitt T., Guo S. (2012). Hepatic suppression of Foxo1 and Foxo3 causes hypoglycemia and hyperlipidemia in mice. Endocrinology.

[92] Eijkelenboom A., Burgering B.M. (2013). FOXOs: Signalling integrators for homeostasis maintenance. Nat. Rev. Mol. Cell Biol..

[93] Leone T.C., Weinheimer C.J., Kelly D.P. (1999). A critical role for the peroxisome proliferator-activated receptor alpha (PPARalpha) in the cellular fasting response: The PPARalpha-null mouse as a model of fatty acid oxidation disorders. Proc. Natl. Acad. Sci. USA.

[94] Kersten S., Seydoux J., Peters J.M., Gonzalez F.J., Desvergne B., Wahli W. (1999). Peroxisome proliferator-activated receptor alpha mediates the adaptive response to fasting. J. Clin. Investig..

[95] Hardie D.G., Carling D., Carlson M. (1998). The AMP-activated/SNF1 protein kinase subfamily: Metabolic sensors of the eukaryotic cell?. Annu. Rev. Biochem..

[96] Carling D. (2004). The AMP-activated protein kinase cascade--a unifying system for energy control. Trends Biochem. Sci..

[97] Jones R.G., Plas D.R., Kubek S., Buzzai M., Mu J., Xu Y., Birnbaum M.J., Thompson C.B. (2005). AMP-activated protein kinase induces a p53-dependent metabolic checkpoint. Mol. Cell.

[98] Molnar Z., Millward A.B., Tse W., Demaine A.G. (2014). p21(WAF1/CIP1) Expression is Differentially Regulated by Metformin and Rapamycin. Int. J. Chronic Dis..

[99] Bungard D., Fuerth B.J., Zeng P.Y., Faubert B., Maas N.L., Viollet B., Carling D., Thompson C.B., Jones R.G., Berger S.L. (2010). Signaling kinase AMPK activates stress-promoted transcription via histone H2B phosphorylation. Science.

[100] Sanli T., Rashid A., Liu C., Harding S., Bristow R.G., Cutz J.C., Singh G., Wright J., Tsakiridis T. (2010). Ionizing radiation activates AMP-activated kinase (AMPK): A target for radiosensitization of human cancer cells. Int. J. Radiat. Oncol. Biol. Phys..

[101] Sanli T., Steinberg G.R., Singh G., Tsakiridis T. (2014). AMP-activated protein kinase (AMPK) beyond metabolism: A novel genomic stress sensor participating in the DNA damage response pathway. Cancer Biol. Ther..

[102] Sapkota G.P., Deak M., Kieloch A., Morrice N., Goodarzi A.A., Smythe C., Shiloh Y., Lees-Miller S.P., Alessi D.R. (2002). Ionizing radiation induces ataxia telangiectasia mutated kinase (ATM)-mediated phosphorylation of LKB1/STK11 at Thr-366. Biochem. J..

[103] Alexander A., Kim J., Walker C.L. (2010). ATM engages the TSC2/mTORC1 signaling node to regulate autophagy. Autophagy.

[104] Seong H.A., Ha H. (2019). Thr55 phosphorylation of p21 by MPK38/MELK ameliorates defects in glucose, lipid, and energy metabolism in diet-induced obese mice. Cell Death Dis..

[105] Fafournoux P., Bruhat A., Jousse C. (2000). Amino acid regulation of gene expression. Biochem. J..

[106] Kimball S.R. (2002). Regulation of global and specific mRNA translation by amino acids. J. Nutr..

[107] Ye J., Kumanova M., Hart L.S., Sloane K., Zhang H., De Panis D.N., Bobrovnikova-Marjon E., Diehl J.A., Ron D., Koumenis C. (2010). The GCN2-ATF4 pathway is critical for tumour cell survival and proliferation in response to nutrient deprivation. EMBO J..

[108] Lehman S.L., Cerniglia G.J., Johannes G.J., Ye J., Ryeom S., Koumenis C. (2015). Translational Upregulation of an Individual p21Cip1 Transcript Variant by GCN2 Regulates Cell Proliferation and Survival under Nutrient Stress. PLoS Genet..

[109] Leung-Pineda V., Pan Y., Chen H., Kilberg M.S. (2004). Induction of p21 and p27 expression by amino acid deprivation of HepG2 human hepatoma cells involves mRNA stabilization. Biochem. J..

[110] Martin-Caballero J., Flores J.M., Garcia-Palencia P., Serrano M. (2001). Tumor susceptibility of p21(Waf1/Cip1)-deficient mice. Cancer Res..

[111] Komiya T., Hosono Y., Hirashima T., Masuda N., Yasumitsu T., Nakagawa K., Kikui M., Ohno A., Fukuoka M., Kawase I. (1997). p21 expression as a predictor for favorable prognosis in squamous cell carcinoma of the lung. Clin. Cancer Res..

[112] Lu X., Toki T., Konishi I., Nikaido T., Fujii S. (1998). Expression of p21WAF1/CIP1 in adenocarcinoma of the uterine cervix: A possible immunohistochemical marker of a favorable prognosis. Cancer.

[113] Ralhan R., Agarwal S., Mathur M., Wasylyk B., Srivastava A. (2000). Association between polymorphism in p21(Waf1/Cip1) cyclin-dependent kinase inhibitor gene and human oral cancer. Clin. Cancer Res..

[114] Kapranos N., Stathopoulos G.P., Manolopoulos L., Kokka E., Papadimitriou C., Bibas A., Yiotakis J., Adamopoulos G. (2001). p53, p21 and p27 protein expression in head and neck cancer and their prognostic value. Anticancer Res..

[115] Roninson I.B. (2002). Oncogenic functions of tumour suppressor p21(Waf1/Cip1/Sdi1): Association with cell senescence and tumour-promoting activities of stromal fibroblasts. Cancer Lett..

[116] Warburg O. (1956). On respiratory impairment in cancer cells. Science.

[117] Warburg O. (1956). On the origin of cancer cells. Science.

[118] Vander Heiden M.G., Cantley L.C., Thompson C.B. (2009). Understanding the Warburg effect: The metabolic requirements of cell proliferation. Science.

[119] Porporato P.E., Filigheddu N., Pedro J.M.B., Kroemer G., Galluzzi L. (2018). Mitochondrial metabolism and cancer. Cell Res..

[120] Teh J.T., Zhu W.L., Newgard C.B., Casey P.J., Wang M. (2019). Respiratory Capacity and Reserve Predict Cell Sensitivity to Mitochondria Inhibitors: Mechanism-Based Markers to Identify Metformin-Responsive Cancers. Mol. Cancer Ther..

[121] Pfleger J., He M., Abdellatif M. (2015). Mitochondrial complex II is a source of the reserve respiratory capacity that is regulated by metabolic sensors and promotes cell survival. Cell Death Dis..

[122] Birsoy K., Possemato R., Lorbeer F.K., Bayraktar E.C., Thiru P., Yucel B., Wang T., Chen W.W., Clish C.B., Sabatini D.M. (2014). Metabolic determinants of cancer cell sensitivity to glucose limitation and biguanides. Nature.

[123] Wheaton W.W., Weinberg S.E., Hamanaka R.B., Soberanes S., Sullivan L.B., Anso E., Glasauer A., Dufour E., Mutlu G.M., Budigner G.S. (2014). Metformin inhibits mitochondrial complex I of cancer cells to reduce tumorigenesis. eLife.

[124] El-Mir M.Y., Nogueira V., Fontaine E., Averet N., Rigoulet M., Leverve X. (2000). Dimethylbiguanide inhibits cell respiration via an indirect effect targeted on the respiratory chain complex I. J. Biol. Chem..

[125] Owen M.R., Doran E., Halestrap A.P. (2000). Evidence that metformin exerts its anti-diabetic effects through inhibition of complex 1 of the mitochondrial respiratory chain. Biochem. J..

[126] Detaille D., Guigas B., Leverve X., Wiernsperger N., Devos P. (2002). Obligatory role of membrane events in the regulatory effect of metformin on the respiratory chain function. Biochem. Pharmacol..

[127] Decensi A., Puntoni M., Goodwin P., Cazzaniga M., Gennari A., Bonanni B., Gandini S. (2010). Metformin and cancer risk in diabetic patients: A systematic review and meta-analysis. Cancer Prev. Res. (Phila.).

[128] Gandini S., Puntoni M., Heckman-Stoddard B.M., Dunn B.K., Ford L., DeCensi A., Szabo E. (2014). Metformin and cancer risk and mortality: A systematic review and meta-analysis taking into account biases and confounders. Cancer Prev. Res. (Phila.).

[129] Chen G., Xu S., Renko K., Derwahl M. (2012). Metformin inhibits growth of thyroid carcinoma cells, suppresses self-renewal of derived cancer stem cells, and potentiates the effect of chemotherapeutic agents. J. Clin. Endocrinol. Metab..

[130] Shank J.J., Yang K., Ghannam J., Cabrera L., Johnston C.J., Reynolds R.K., Buckanovich R.J. (2012). Metformin targets ovarian cancer stem cells in vitro and in vivo. Gynecol. Oncol..

[131] Weinberg S.E., Chandel N.S. (2015). Targeting mitochondria metabolism for cancer therapy. Nat. Chem. Biol..

[132] Hsu M.C., Hung W.C. (2018). Pyruvate kinase M2 fuels multiple aspects of cancer cells: From cellular metabolism, transcriptional regulation to extracellular signaling. Mol. Cancer.

[133] Bardella C., Pollard P.J., Tomlinson I. (2011). SDH mutations in cancer. Biochim. Biophys. Acta.

[134] Reitman Z.J., Yan H. (2010). Isocitrate dehydrogenase 1 and 2 mutations in cancer: Alterations at a crossroads of cellular metabolism. J. Natl. Cancer Inst..

[135] Bos J.L. (1989). Ras oncogenes in human cancer: A review. Cancer Res..

[136] Nicholson R.I., Gee J.M., Harper M.E. (2001). EGFR and cancer prognosis. Eur. J. Cancer.

[137] Davies H., Bignell G.R., Cox C., Stephens P., Edkins S., Clegg S., Teague J., Woffendin H., Garnett M.J., Bottomley W. (2002). Mutations of the BRAF gene in human cancer. Nature.

[138] Kohl N.E., Ruley H.E. (1987). Role of c-myc in the transformation of REF52 cells by viral and cellular oncogenes. Oncogene.

[139] Ridley A.J., Paterson H.F., Noble M., Land H. (1988). Ras-mediated cell cycle arrest is altered by nuclear oncogenes to induce Schwann cell transformation. EMBO J..

[140] Serrano M., Lin A.W., McCurrach M.E., Beach D., Lowe S.W. (1997). Oncogenic ras provokes premature cell senescence associated with accumulation of p53 and p16INK4a. Cell.

[141] Olson M.F., Paterson H.F., Marshall C.J. (1998). Signals from Ras and Rho GTPases interact to regulate expression of p21Waf1/Cip1. Nature.

[142] Manu K.A., Chai T.F., Teh J.T., Zhu W.L., Casey P.J., Wang M. (2017). Inhibition of Isoprenylcysteine Carboxylmethyltransferase Induces Cell-Cycle Arrest and Apoptosis through p21 and p21-Regulated BNIP3 Induction in Pancreatic Cancer. Mol. Cancer Ther..

[143] Bergo M.O., Gavino B.J., Hong C., Beigneux A.P., McMahon M., Casey P.J., Young S.G. (2004). Inactivation of Icmt inhibits transformation by oncogenic K-Ras and B-Raf. J. Clin. Investig..

[144] Wang M., Tan W., Zhou J., Leow J., Go M., Lee H.S., Casey P.J. (2008). A small molecule inhibitor of isoprenylcysteine carboxymethyltransferase induces autophagic cell death in PC3 prostate cancer cells. J. Biol. Chem..

[145] Wang M., Hossain M.S., Tan W., Coolman B., Zhou J., Liu S., Casey P.J. (2010). Inhibition of isoprenylcysteine carboxylmethyltransferase induces autophagic-dependent apoptosis and impairs tumor growth. Oncogene.

[146] Lau H.Y., Ramanujulu P.M., Guo D., Yang T., Wirawan M., Casey P.J., Go M.L., Wang M. (2014). An improved isoprenylcysteine carboxylmethyltransferase inhibitor induces cancer cell death and attenuates tumor growth in vivo. Cancer Biol. Ther..

[147] Teh J.T., Zhu W.L., Ilkayeva O.R., Li Y., Gooding J., Casey P.J., Summers S.A., Newgard C.B., Wang M. (2015). Isoprenylcysteine carboxylmethyltransferase regulates mitochondrial respiration and cancer cell metabolism. Oncogene.

[148] Winter-Vann A.M., Baron R.A., Wong W., dela Cruz J., York J.D., Gooden D.M., Bergo M.O., Young S.G., Toone E.J., Casey P.J. (2005). A small-molecule inhibitor of isoprenylcysteine carboxyl methyltransferase with antitumor activity in cancer cells. Proc. Natl. Acad. Sci. USA.

[149] Ramanujulu P.M., Yang T., Yap S.Q., Wong F.C., Casey P.J., Wang M., Go M.L. (2013). Functionalized indoleamines as potent, drug-like inhibitors of isoprenylcysteine carboxyl methyltransferase (Icmt). Eur. J. Med. Chem..

[150] Fujiwara K., Daido S., Yamamoto A., Kobayashi R., Yokoyama T., Aoki H., Iwado E., Shinojima N., Kondo Y., Kondo S. (2008). Pivotal role of the cyclin-dependent kinase inhibitor p21WAF1/CIP1 in apoptosis and autophagy. J. Biol. Chem..

[151] Capparelli C., Chiavarina B., Whitaker-Menezes D., Pestell T.G., Pestell R.G., Hulit J., Ando S., Howell A., Martinez-Outschoorn U.E., Sotgia F. (2012). CDK inhibitors (p16/p19/p21) induce senescence and autophagy in cancer-associated fibroblasts, "fueling" tumor growth via paracrine interactions, without an increase in neo-angiogenesis. Cell Cycle.

[152] Levine B., Klionsky D.J. (2004). Development by self-digestion: Molecular mechanisms and biological functions of autophagy. Dev. Cell.

[153] Mizushima N. (2007). Autophagy: Process and function. Genes Dev..

[154] Altman B.J., Rathmell J.C. (2012). Metabolic stress in autophagy and cell death pathways. Cold Spring Harb Perspect. Biol..

[155] Kaur J., Debnath J. (2015). Autophagy at the crossroads of catabolism and anabolism. Nat. Rev. Mol. Cell Biol..

[156] Mortimore G.E., Poso A.R. (1987). Intracellular protein catabolism and its control during nutrient deprivation and supply. Annu. Rev. Nutr..

[157] Rubinsztein D.C., Gestwicki J.E., Murphy L.O., Klionsky D.J. (2007). Potential therapeutic applications of autophagy. Nat. Rev. Drug Discov..

[158] Levine B., Kroemer G. (2008). Autophagy in the pathogenesis of disease. Cell.

[159] Chen N., Karantza-Wadsworth V. (2009). Role and regulation of autophagy in cancer. Biochim. Biophys. Acta.

[160] Chen S., Rehman S.K., Zhang W., Wen A., Yao L., Zhang J. (2010). Autophagy is a therapeutic target in anticancer drug resistance. Biochim. Biophys. Acta.

[161] Gozuacik D., Kimchi A. (2007). Autophagy and cell death. Curr. Top. Dev. Biol..

[162] Chen G., Ray R., Dubik D., Shi L., Cizeau J., Bleackley R.C., Saxena S., Gietz R.D., Greenberg A.H. (1997). The E1B 19K/Bcl-2-binding protein Nip3 is a dimeric mitochondrial protein that activates apoptosis. J. Exp. Med..

[163] Adams J.M., Cory S. (1998). The Bcl-2 protein family: Arbiters of cell survival. Science.

[164] Bruick R.K. (2000). Expression of the gene encoding the proapoptotic Nip3 protein is induced by hypoxia. Proc. Natl. Acad. Sci. USA.

[165] Ray R., Chen G., Vande Velde C., Cizeau J., Park J.H., Reed J.C., Gietz R.D., Greenberg A.H. (2000). BNIP3 heterodimerizes with Bcl-2/Bcl-X(L) and induces cell death independent of a Bcl-2 homology 3 (BH3) domain at both mitochondrial and nonmitochondrial sites. J. Biol. Chem..

[166] Yasuda M., Theodorakis P., Subramanian T., Chinnadurai G. (1998). Adenovirus E1B-19K/BCL-2 interacting protein BNIP3 contains a BH3 domain and a mitochondrial targeting sequence. J. Biol. Chem..

[167] Erkan M., Kleeff J., Esposito I., Giese T., Ketterer K., Buchler M.W., Giese N.A., Friess H. (2005). Loss of BNIP3 expression is a late event in pancreatic cancer contributing to chemoresistance and worsened prognosis. Oncogene.

[168] Akada M., Crnogorac-Jurcevic T., Lattimore S., Mahon P., Lopes R., Sunamura M., Matsuno S., Lemoine N.R. (2005). Intrinsic chemoresistance to gemcitabine is associated with decreased expression of BNIP3 in pancreatic cancer. Clin. Cancer Res..

[169] Okami J., Simeone D.M., Logsdon C.D. (2004). Silencing of the hypoxia-inducible cell death protein BNIP3 in pancreatic cancer. Cancer Res..

[170] Hamacher-Brady A., Brady N.R., Logue S.E., Sayen M.R., Jinno M., Kirshenbaum L.A., Gottlieb R.A., Gustafsson A.B. (2007). Response to myocardial ischemia/reperfusion injury involves Bnip3 and autophagy. Cell Death Differ..

[171] Tracy K., Dibling B.C., Spike B.T., Knabb J.R., Schumacker P., Macleod K.F. (2007). BNIP3 is an RB/E2F target gene required for hypoxia-induced autophagy. Mol. Cell. Biol..

[172] Crnogorac-Jurcevic T., Efthimiou E., Nielsen T., Loader J., Terris B., Stamp G., Baron A., Scarpa A., Lemoine N.R. (2002). Expression profiling of microdissected pancreatic adenocarcinomas. Oncogene.

[173] Friess H., Ding J., Kleeff J., Fenkell L., Rosinski J.A., Guweidhi A., Reidhaar-Olson J.F., Korc M., Hammer J., Buchler M.W. (2003). Microarray-based identification of differentially expressed growth- and metastasis-associated genes in pancreatic cancer. Cell. Mol. Life Sci..

